# Association between Torque teno virus-DNA plasma loads and post-transplantation diabetes mellitus in the first year after kidney transplantation

**DOI:** 10.1186/s12882-026-05192-6

**Published:** 2026-07-09

**Authors:** Felix Eisinger, Charikleia Gkioule, Anja Schork, Silvio Nadalin, Andreas Birkenfeld, Thomas Iftner, Nils Heyne, Martina Guthoff, Tina Ganzenmueller

**Affiliations:** 1 Internal Medicine IV, Department of Diabetology, Endocrinology and Nephrology, University of Tübingen, Tübingen, Germany; 2 Institute for Diabetes Research and Metabolic Diseases (IDM) of Helmholtz Munich, University of Tübingen, Tübingen, Germany; 3 German Center for Diabetes Research (DZD), Tübingen, Germany; 4 Institute of Medical Virology, University Hospital Tübingen, Elfriede-Aulhorn-Str. 6, 72076 Tübingen, Germany; 5 German Center for Infection Research (DZIF) at the University Hospital Tübingen, Tübingen, Germany; 6 Department of General and Transplant Surgery, University of Tübingen, Tübingen, Germany; 7 Nephrological Center Villingen-Schwenningen, Villingen-Schwenningen, Germany

**Keywords:** Post-transplantation diabetes mellitus, Torque teno virus, Biomarker, Immunosuppression

## Abstract

**Background:**

Torque teno virus (TTV) DNAemia is a surrogate marker of immune function in renal transplant recipients (RTRs), usually peaking around 3 months after transplantation. Post-transplantation diabetes mellitus (PTDM), a condition with impaired immune response, is common after renal transplantation, but its relationship with TTV replication remains unclear.

**Methods:**

In this retrospective study, TTV-DNA loads were analysed in 303 plasma samples from 93 RTRs collected shortly after transplantation and at 3, 6, and 12 months. Clinical and laboratory parameters, including glycaemic status, were assessed. 17 patients (18.3%) developed PTDM.

**Results:**

As expected, TTV-DNA plasma loads peaked at 3 and 6 months in the cohort. Upon multivariate analysis, time after transplantation (*p* < 0.001), tacrolimus trough levels, (*p* = 0.001), and HbA1c (*p* = 0.012) were associated with TTV-DNA plasma loads. Patients with PTDM, but not with pre-existing diabetes mellitus, showed significantly higher TTV-DNAemia at 3 months (*p* = 0.03) and 6 months (*p* = 0.01) after transplantation compared to non-diabetic patients. No significant group differences were observed for cumulative prednisolone dose, tacrolimus trough levels and induction therapy. Although patients with PTDM were older, age contributed only marginally to TTV-DNAemia. Opportunistic viral infections tended to occur more frequently in patients with PTDM.

**Conclusions:**

In conclusion, PTDM but not pre-existing diabetes was associated with elevated TTV-DNA plasma loads early after transplantation in this retrospective cohort analysis. Patients with PTDM also showed a trend towards more opportunistic infections, although this observation should be interpreted cautiously given the small number of events. These findings suggest a potential association between PTDM and altered immune regulation in RTRs that warrants further investigation.

**Supplementary Information:**

The online version contains supplementary material available at 10.1186/s12882-026-05192-6.

## Background

Maintaining the optimal balance between over- and under-immunosuppression remains a central challenge after kidney transplantation. Despite therapeutic drug monitoring, clinicians still lack a reliable biomarker that reflects immune function. In this context, Torque teno virus (TTV) has emerged as a promising candidate. Torque teno viruses are non-pathogenic DNA-viruses and belong to the *Anelloviridae* family [1]. They are ubiquitous in most human bodily fluids, can be detected in 90% of the general population and in nearly all patients after solid organ transplantation [2]. Together with the less frequent Torque teno mini viruses and Torque teno midi viruses, TTV represent a major part of the human anellome, and thus the plasma human virome [3–5]. In renal transplant recipients (RTRs), TTV plasma loads usually peak around 60 to 90 days after transplantation [6]. In this population, several studies indicate that TTV-DNA plasma load determined by quantitative real-time PCR correlates with both over- and under-immunosuppression, making TTV-DNAemia a potential surrogate marker of immune function [7–10]. Accordingly, higher TTV-DNA plasma loads have been associated with opportunistic infections, whereas lower TTV-DNA load levels have correlated with rejection episodes [11, 12]. Nevertheless, current evidence remains heterogeneous. Most available studies were conducted within the first year after transplantation, and clinically validated TTV cut-offs are still lacking, partly due to the use of different assays for TTV quantification [9]. Furthermore, associations between low TTV-DNA plasma loads and rejection episodes appear more consistent than findings regarding infectious complications, which have shown greater variability across cohorts and study designs [13]. Nevertheless, the concept of TTV-DNA plasma load as a potential biomarker of immune status remains promising and has attracted increasing interest in recent years.

Several factors have been identified to influence the magnitude of TTV-DNAemia in RTRs, with immunosuppressant trough levels and composition of immunosuppressive regimens being the main determinants [7]. Tacrolimus, as to date the most investigated drug in this context, appears to be one of the key factors influencing TTV-DNA plasma loads, but also corticosteroids, often administered as pulse therapy for mitigation of ischemia-reperfusion damage and rejection, have been associated with higher TTV-DNA plasma loads [14, 15] as well as induction therapy. Patients receiving anti-thymocyte globulin (ATG) for induction show higher TTV plasma loads 3 months after transplantation compared to those receiving basiliximab [16]. Beyond these pharmacological factors, patient-specific factors including age and body mass index (BMI) have been associated with TTV-DNAemia dynamics [15, 17].

Given that TTV-DNA plasma loads reflect the overall degree of immunosuppression, metabolic disturbances that impair immune function may also influence TTV replication dynamics. Post-transplant diabetes mellitus (PTDM) is a common metabolic complication after transplantation, affecting approximately 10–20% of RTRs [18]. While the underlying pathophysiology of PTDM is not fully understood, multiple risk factors − such as immunosuppressive therapy (corticosteroids, calcineurin inhibitors, possibly mTOR inhibitors), pre-diabetic status, family history, obesity, age, infections, rejection episodes and genetic predisposition − have been identified [19]. Although PTDM is a highly dynamic condition with up to 30% of early PTDM cases resolving over time [18, 20], its onset is associated with poorer transplant outcomes, including increased cardiovascular mortality and a higher risk of infections [21, 22]. As both hyperglycemia and insulin resistance are known to impair immune response — including neutrophil, macrophage and NK-cell function, as well as cytokine production [23, 24] — PTDM may also affect immune function in RTRs and thereby influence TTV-DNAemia. In the general population, data on diabetes mellitus (DM) and TTV-DNA plasma loads are limited, but reports suggest a slightly higher TTV prevalence in individuals with type 2 DM [25, 26]. To our knowledge, no corresponding data are available for transplant recipients. The present study aims to explore the potential association between PTDM and TTV-DNAemia after kidney transplantation, adjusting for relevant confounders such as immunosuppressive therapy and patient-related factors.

## Methods

### Study outline and patient cohort

In this retrospective study, 303 archived plasma samples from 93 adult patients who underwent kidney transplantation at the University Hospital Tübingen between January 2020 and December 2023 were analyzed to quantify TTV-DNA plasma loads. Details of patient screening and selection are shown in Supplementary Figure S1. Clinical data — including presence and subtype of DM, PTDM, immunosuppressive medication, episodes of allograft rejection, opportunistic infections, and relevant laboratory parameters — were retrieved from medical records to identify factors influencing TTV-DNAemia.

Plasma samples were originally sent to the Institute for Medical Virology for routine post-transplant PCR monitoring of clinically relevant viral reactivations (e.g., BK virus (BKV) or cytomegalovirus (CMV)). Remnant plasma samples (archived at -20 °C) obtained at predefined time points after transplantation were selected. Baseline samples were collected at a median [IQR] of 4.0 [2.5–6.0] days, with follow-up samples at 3 months (90.0 [86.0–100.5] days), 6 months (185.0 [173.8–198.3] days), and 12 months (368.5 [350.5–379.0] days) after transplantation. Only RTRs with an available baseline sample and at least one available follow-up plasma samples were included in the final analysis.

## Quantification of TTV-DNA plasma load using real-time PCR

DNA was extracted from plasma samples using the EMAG platform (bioMérieux) according to the manufacturer’s instructions. Quantification of TTV-DNA plasma load levels (TTV-DNAemia) was performed by quantitative real-time PCR by using the CE-certified TTV R-GENE© kit (bioMérieux). Real-time PCR was carried out on a LightCycler 480 system (Roche) following the manufacturer’s protocol. For statistical analyses, samples with viral loads below the lower limit of quantification (LLOQ, < 250 copies/ml) were assigned a value of 250 copies/ml prior to log10 transformation.

## Assessment of post-transplantation diabetes mellitus (PTDM)

According to international guidelines, the diagnostic criteria for PTDM include: (1) classic symptoms of hyperglycemia and a random plasma glucose ≥ 200 mg/dl (11.1 mmol/l), (2) fasting plasma glucose ≥ 126 mg/dl (7.0 mmol/l), (3) oral glucose tolerance test (OGTT) with a 2 h plasma glucose ≥ 200 mg/dl (11.1 mmol/l), or (4) HbA1c ≥ 6.5% (48 mmol/mol) [27]. Although recent international consensus statements recommend the OGTT as the preferred method for PTDM detection [28], its use in clinical routine is often limited due to logistical constraints. In this retrospective study, HbA1c and fasting plasma glucose were used as diagnostic criteria due to consistent data availability. In order to account for fluctuations of immunosuppression, kidney function and hemoglobin levels in the early post-transplant period and the limited reliability of HbA1c during this time [29], only HbA1c measurements obtained at least 3 months after transplantation were considered for PTDM diagnosis. Patients with pre-existing DM at the time of transplantation were analysed separately. For comparisons of patient characteristics, including demographic variables, immunosuppressive exposure (tacrolimus trough levels and prednisolone dose), and follow-up laboratory parameters, all patients who developed PTDM during follow-up were assigned to the PTDM group to allow characterization of the overall PTDM cohort. For analyses of TTV-DNAemia at specific time points (3, 6, and 12 months after transplantation), patients were assigned to the PTDM group only if the diagnosis had been established at or before the respective time point to avoid retrospective group assignment.

## Assessment of opportunistic infections

The following infections were considered as clinically relevant opportunistic or immunosuppression-associated infections in our patient cohort: CMV, BKV, Epstein-Barr Virus (EBV), *Aspergillus* spp., *Candida* spp., *Pneumocystis jirovecii*, and chronic hepatitis E virus infections. Diagnosis and monitoring of viral reactivations and/or diseases were performed as part of clinical routine PCR testing. For CMV and EBV reactivation, a viral load threshold of ≥ 500 copies/ml plasma (corresponding to the assays´ LLOQ) was defined as clinically relevant viremia, for BKV reactivations a threshold of ≥ 1,000 copies/ml plasma was applied [30]. Infections were counted once per patient.

### Ethics approval and consent to participate

The study was approved by the local Ethics Committee of the University Hospital Tübingen (number 211/2023BO2). The requirement for written informed consent was waived, due to the study’s retrospective nature, and because it utilized remnant plasma samples collected for routine diagnostic purposes together with pseudonymized clinical data. This study was conducted in accordance with the principles of the Declaration of Helsinki and complied with all relevant institutional and national guidelines and regulations.

### Statistics

Statistical calculations were performed using GraphPad Prism (version 10.6.1 for Windows) and IBM SPSS Statistics (version 31.0.0.0). Quantitative data are presented as median with interquartile range unless otherwise stated. Continuous variables were compared using the Mann–Whitney U test or the Kruskal–Wallis test, as appropriate. A chi-square test was used to compare categorical variables, such as the presence of PTDM and the occurrence of opportunistic infections. In order to evaluate longitudinal determinants of TTV-DNA plasma loads, a linear mixed model was fitted with TTV-DNA plasma load as the dependent variable. All TTV-DNA plasma load measurements (time of transplantation (baseline) and 3, 6, and 12 months after transplantation) were included in the model to capture the full post-transplant course of TTV-DNAemia. Time after transplantation, tacrolimus trough level, and HbA1c were entered as fixed effects. These variables were selected as potentially relevant determinants of TTV-DNAemia after kidney transplantation. Additional clinically relevant covariates, including age, sex, and steroid dose, were explored in alternative model specifications. However, given the limited sample size, inclusion of additional fixed effects increased model complexity without improving model fit according to Akaike's Information Criterion (AIC) and were therefore not retained in the final model. The random-effects structure consisted of a patient-specific random intercept with a variance components covariance structure. Furthermore, repeated observations were grouped by patient. Missing observations were not imputed, and patients with incomplete data were included under the assumption that data were missing at random. Given the exploratory nature of the study, no formal adjustment for multiple testing was performed. Statistical significance was defined as a *p*-value <0.05.

## Results

### Patient characteristics

The median age of the study population was 55 [40 – 65] years, and 61.3% (57/93) of patients were male. Overall, 17 out of 93 patients (18.3%) developed PTDM during the study period, while 11 patients (11.9%) had pre-existing DM (type 1: 2/11, type 2: 9/11). Most cases of PTDM occurred within the first 6 months after kidney transplantation, with a median onset of 110 [87 – 201] days. The median BMI was 24 [21.2 – 27.3] kg/m2. Additional patient characteristics are presented in Table 1. Patients with pre-existing DM showed significantly higher HbA1c levels (*p*=0.003) and fasting plasma glucose levels (*p*=0.016) at 12 months compared to patients with PTDM (Supplementary Table 1), whereas no significant differences in glycaemic parameters between the groups were observed at 3 or 6 months. 

### Overall TTV-DNA plasma loads kinetics following kidney transplantation

**Fig. 1:**
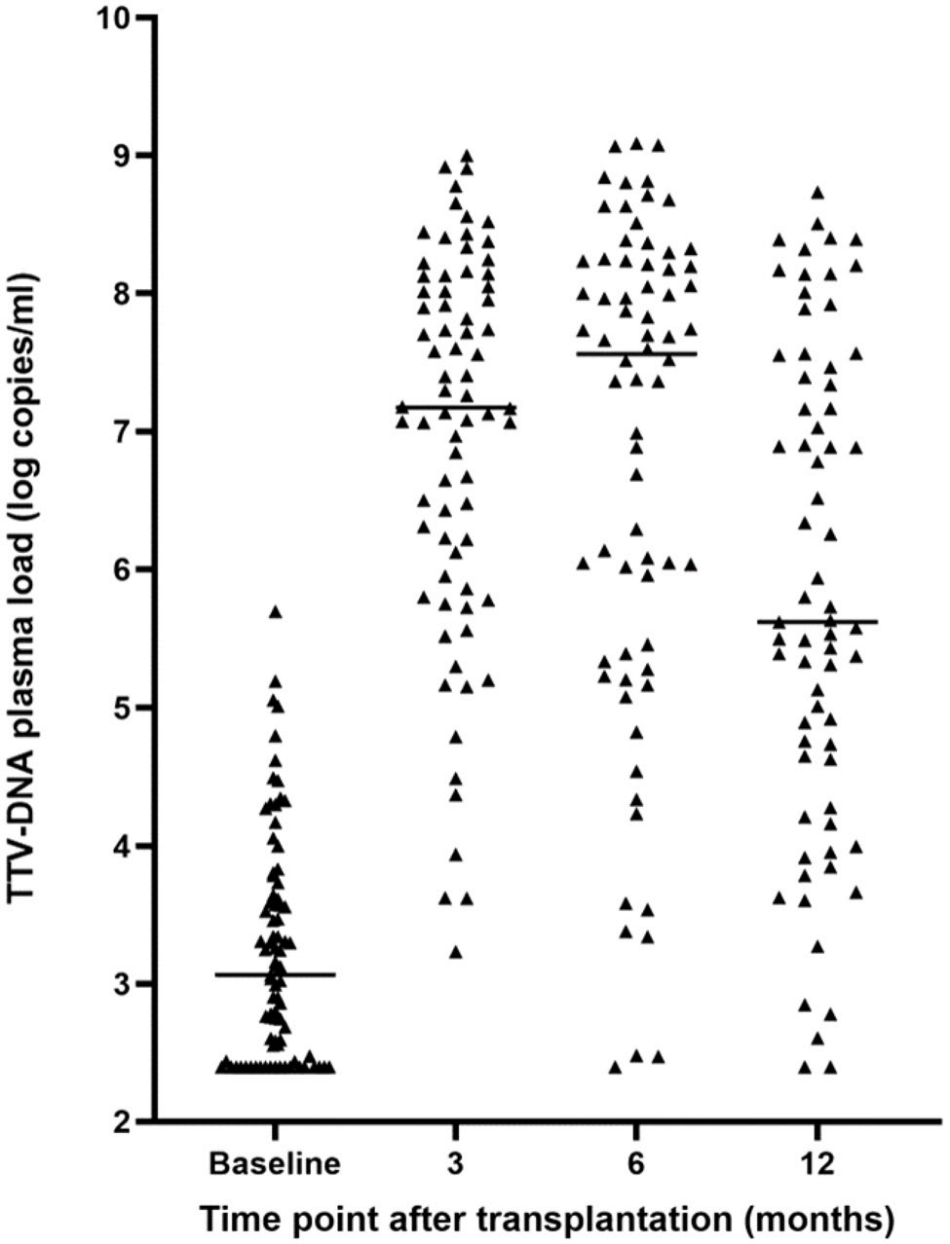
Dynamics of TTV-DNA plasma load over time. Illustration of TTV-DNA plasma loads at baseline and 3, 6, and 12 months after kidney transplantation in the complete study population (*n* = 93). Individual symbols represent TTV-DNA plasma loads expressed as log₁₀ copies/mL. Differences of the median TTV-DNAemia load levels between time points were analyzed using the Kruskal–Wallis test

TTV-DNA plasma loads (TTV-DNAemia) were successfully determined in 303 plasma samples obtained longitudinally from the 93 RTR included in the study. The median TTV-DNA plasma load increased significantly (p<0.001) from 3.1 [2.4 – 3.6] log10 copies/ml at baseline to 7.2 [5.9 – 8.1] log10 copies/ml after 3 months. After 6 months, median levels reached 7.6 [5.4 – 8.2] log10 copies/ml followed by a significant (*p*=0.007) decrease to 5.6 [4.5 – 7.4] log10 copies/ml after 12 months. The distribution of TTV-DNA plasma loads over time is shown in Figure 1 and Supplementary Figure S2. Overall, a total of 31 samples (10.2%) showed TTV-DNA plasma loads below the LLOQ.

### Determinants of TTV-DNAemia: linear mixed-effects model

A linear mixed-effects model was used to identify factors associated with TTV-DNAemia in our RTR cohort (Table 2). Time post-transplantation showed a significant association with higher TTV-DNA plasma loads (estimate 0.21, 95% CI 0.15–0.26, p < 0.001). Tacrolimus trough levels (estimate 0.10, 95% CI 0.04–0.16, p = 0.001) and HbA1c values (estimate 0.37, 95% CI 0.08–0.65, p = 0.012) were also positively associated with TTV-DNAemia. 

### TTV-DNA plasma loads in the context of PTDM

**Fig. 2:**
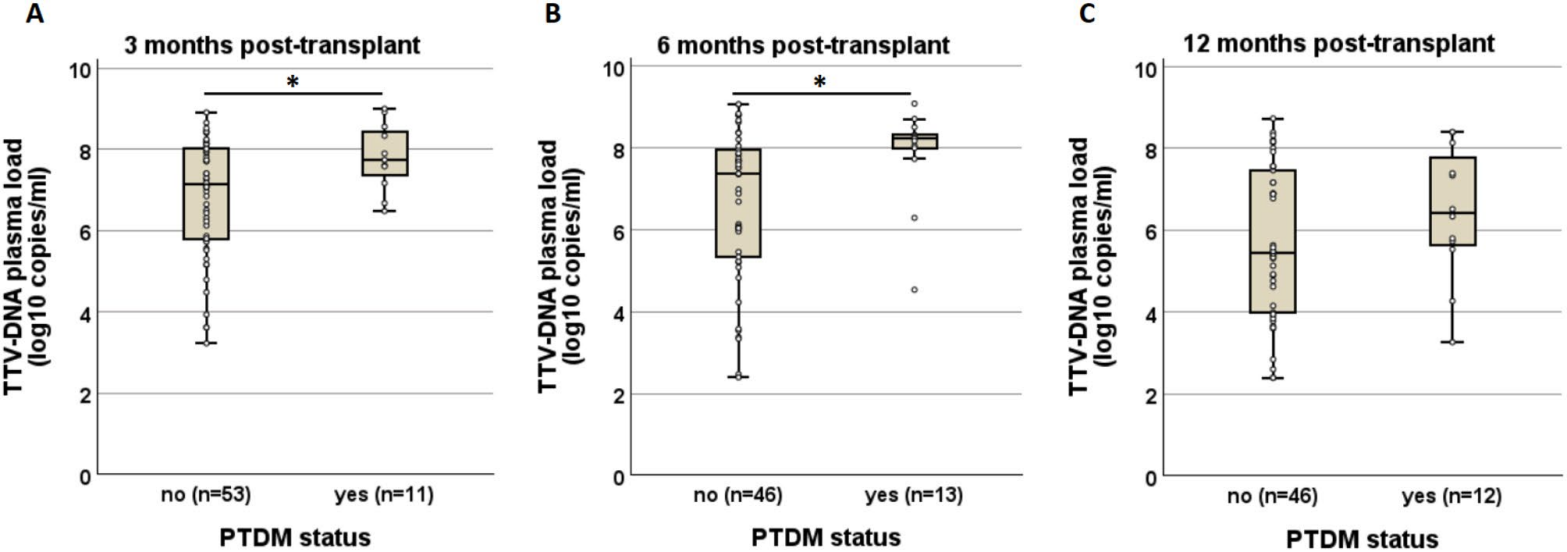
**A**-**C**. Torque teno virus (TTV)-DNA plasma load in patients with and without post-transplant diabetes mellitus (PTDM) at 3 months (**A**), 6 months (**B**), and 12 months (**C**) after kidney transplantation. TTV-DNA plasma loads are shown as log₁₀ copies/ml. Patients were classified as PTDM if the diagnosis had been established at or before the respective time point, and those with pre-existing diabetes mellitus were excluded from this analysis. Boxplots represent median values and interquartile ranges, while individual data points depict the distribution of available measurements. The number of available samples for each group is indicated below the respective boxplots. Differences in TTV-DNAemia levels between groups were assessed using the Mann–Whitney U test

Given the association between HbA1c values and TTV-DNAemia in the linear mixed model, TTV-DNA plasma loads values were subsequently analyzed based on PTDM status at different time points (Figure 2). Three months after transplantation, patients with PTDM showed significantly higher TTV-DNA plasma loads than those without PTDM (7.7 [7.2 – 8.6] vs. 7.1 [5.8 – 8.0] log10 copies/ml, p=0.033). At 6 months, the median TTV-DNA plasma load was significantly higher with 8.2 [8.0–8.3] log₁₀ copies/ml in patients with PTDM compared to 7.4 [5.3–8.0] log₁₀ copies/ml in those without PTDM (*p* = 0.011). At 12 months, median TTV-DNA plasma loads did not differ significantly between groups, with 6.4 [5.6–7.8] log₁₀ copies/ml in the PTDM group and 5.5 [4.0–7.5] log₁₀ copies/ml in the non-PTDM group (*p* = 0.167). In patients with pre-existing type 1 or type 2 DM, median TTV-DNA plasma loads did not differ significantly across all selected time points from those in patients without DM (Supplementary Figure S3). In addition, patients with PTDM showed significant higher median TTV-DNA plasma loads at six months after transplantation than patients with pre-existing DM (8.2 [8.0–8.3] vs. 6.1 [5.3 – 8.0] log10 copies/ml, p=0.03) 

### Assessment of potential confounding factors 

To determine whether additional clinical variables affected the association between PTDM status and TTV-DNAemia, further analyses were conducted including the association with cumulative prednisolone dose, tacrolimus trough levels, age, BMI and induction therapy. Characteristics of the PTDM and non-PTDM groups for these variables are presented in (Table 1). Neither tacrolimus trough levels nor cumulative prednisolone dose differed significantly between the groups at any of the examined time points. In a multiple linear regression model, the PTDM status remained significantly associated with TTV-DNA plasma loads at 3 and 6 months after inclusion of tacrolimus trough levels (Supplementary Table 2). Regarding induction therapy and BMI, there were no significant differences between the PTDM and non-PTDM groups (Table 1). Finally, overall age of patients with PTDM was significantly higher than of those without PTDM (62 [57 – 66] vs. 51 [36 – 62] years, p=0.001). Since age-related immunosenescence may influence TTV replication, we performed an additional exploratory linear regression analysis to estimate the potential contribution of age to the observed differences in TTV-DNAemia between the groups. However, the effects of PTDM on TTV-DNA plasma loads exceeded the estimated effects of age at all time points (Supplementary Table 3). 

### PTDM status and opportunistic infections

Overall, 24 out of 93 patients (25.8%) developed an opportunistic infection within the first year after transplantation (Table 1). The median time to onset of the first obeserved infection episode was 112 [80 – 189] days. The majority of events were BKV (12.9% ) or CMV reactivations (7.5%). Patients with PTDM had slightly more opportunistic infection episodes (35.3% (6/17)) compared to patients without DM (23.1% (15/65)). When stratifying the distribution of BKV and CMV infection cases by PTDM status, viral reactivations occurred in 29.4% (5/17) of patients with PTDM and in 18.5% (12/65) of those without DM. However, these differences did not reach statistical significance (Table 1). 

### Discussion

In this analysis of 93 renal transplant recipients, we detected – to our knowledge for the first time - a potential association between PTDM and TTV-DNAemia: patients who developed PTDM exhibited significantly higher TTV-DNA plasma loads compared to non-diabetic patients three and six months after transplantation. Furthermore, we observed a higher frequency of infections in patients with PTDM than in non-diabetic patients, although this difference did not reach statistical significance. While our findings should be interpreted with caution due to the moderate sample size and the exploratory nature of the analysis, they suggest a potential association between PTDM, altered immune status and TTV-DNAemia in kidney transplant recipients that warrants further validation in external cohorts.

The association between PTDM and elevated TTV-DNA plasma load remained robust after adjusting for key potential confounders, including tacrolimus trough levels, corticosteroid dose, and the type of induction therapy. These findings support a potential link beyond major treatment-related factors, although the underlying mechanisms remain to be clarified. Furthermore, while patients with PTDM were significantly older, a secondary analysis demonstrated that the relationship between PTDM and TTV-DNA plasma loads could not be explained by age alone. This interpretation is supported by data on TTV-DNAemia in 1000 healthy blood donors, where Focosi and colleagues showed that ageing accounted for only minor increases of TTV-DNAemia in this population [31]. Although TTV-DNA plasma load might be a marker of immunosenescence, longitudinal studies are needed to further clarify the underlying mechanisms [32].

The association between PTDM and TTV-DNAemia observed in our study may be explained by underlying -potentially shared- causes. One potential contributor might be hyperglycemia-induced immune cell dysfunction. Studies in patients with type 2 DM have demonstrated reduced T-cell numbers, impaired pathogen-specific memory CD4⁺ T-cell responses, and decreased T-cell–mediated cytotoxicity in this cohort [33–35]. In a murine model of type 2 DM, hyperglycemia — but not hyperinsulinemia — was associated with a diminished CD8⁺ T-cell response to viral infections [36]. These functional alterations may be driven by hyperglycemia-related mechanisms such as protein glycation and increased oxidative stress, which can adversely affect immune cell signaling and viability [37, 38].

Although the precise pathways regulating immunological control of TTV are not fully understood, accumulating evidence suggests a central role for T-cell–mediated mechanisms. In the general population, higher TTV loads have been associated with lower CD4⁺ T-cell counts and a reduced CD4/CD8 ratio [39]. Against this background, PTDM-associated T-cell dysfunction might contribute to the elevated TTV-DNA plasma loads observed in our cohort. Interestingly, we did not find increased TTV-DNA plasma loads in patients with pre-existing DM, despite significantly elevated glucose levels in this group. While the cohort of patients with pre-existing diabetes was relatively small and heterogeneous, potentially limiting the ability to detect meaningful differences, this finding suggests that hyperglycemia alone may not be sufficient to fully explain the association between PTDM and TTV replication. Other aspects of glucose metabolism, such as hyperinsulinemia, may represent additional mechanisms and cannot be excluded.

Beyond hyperglycemia, PTDM is frequently accompanied by a proinflammatory metabolic milieu characterized by increased levels of inflammatory cytokines and oxidative stress [40]. In a retrospective study in RTRs, inflammation-related biomarkers such as tumour necrosis factor type 1 or endothelial protein C receptor have been reported to be significantly associated with the presence of PTDM [41]. This inflammatory state might compromise effective antiviral immune surveillance, thereby facilitating increased TTV replication. Supporting this hypothesis, recent data have demonstrated an association between elevated pro-inflammatory cytokine levels and increased TTV-DNA plasma loads [39]. In the context of PTDM, inflammation might act synergistically with metabolic dysregulation to exacerbate immune dysfunction in the early post-transplant period.

Another contributing factor in the relationship between PTDM and TTV-DNAemia may be the presence of CMV reactivations, as reported by previous studies, which found an association of CMV viremia with both the occurrence of PTDM and higher TTV-DNA plasma loads [42, 43]. In line with these findings, we observed a higher incidence of CMV-DNAemia and higher TTV plasma loads in patients with PTDM compared to those with pre-existing DM or without DM. The exact underlying mechanisms of this relationship is unclear. Both TTV- and CMV-DNAemia could simply be a marker of over-immunosuppression. Furthermore, CMV is known to exert multiple effects on the immune system via its immunoevasive functions. The virus impairs the activation and proliferation of T-cells, thereby contributing to a state of functional immunosuppression [44, 45]. CMV infection increases the release of pro-inflammatory cytokines and promotes inflammasome activation, which may enhance systemic inflammatory response [46]. In the context of our findings, CMV-induced immune modulation and inflammation may represent one possible link between PTDM and TTV-DNAemia. However, it is also plausible that an increased net state of immunosuppression could promote both CMV reactivation and the development of PTDM or that this effect is confounded by higher CMV seropositivity rates in older patients.

In general, PTDM is a multifactorial condition influenced by a wide range of factors, including genetic predisposition, immunosuppressive therapy, inflammatory burden, infectious complications, preexisting impaired glucose tolerance, age, and body weight [19]. Many of these factors do not only affect glucose metabolism but also modulate immune function. This large number of potential confounders complicates causal interpretation. Thus, it cannot be conclusively determined whether PTDM-driven immune alterations directly influence TTV plasma loads or whether increases of PTDM incidence and of TTV-DNAemia levels arise from one or more shared underlying covariates. Although no statistically significant differences between groups were observed for several additional variables assessed in our cohort, clinically meaningful differences cannot be completely excluded, particularly given the relatively small sample size. Therefore, factors such as rejection episodes, steroid pulse therapy and overall immunosuppressive burden may still represent potential confounders and could have contributed to the observed association between PTDM and elevated TTV-DNAemia.

Interestingly, a higher frequency of infectious complications, particularly CMV or BKV reactivation, was observed in the PTDM group. However, given the limited number of events and the influence of multiple co-factors, these findings should be interpreted cautiously and do not allow general conclusions regarding a direct association between PTDM, TTV-DNA plasma loads, and infectious outcomes. Furthermore, the temporal relationship between PTDM onset and infectious events was not systematically assessed in the present study. Overall, it is more difficult to show a strict association of TTV-DNA plasma loads with infection outcomes, due to multiple co-factors [13]. Nevertheless, an elevated rate of viral reactivation in patients with PTDM seems biologically plausible and is in line with many studies reporting a higher incidence of opportunistic infections together with higher TTV-DNA plasma loads [7]. Given that PTDM has been associated with adverse clinical outcomes such as higher infection rates and delayed graft function [47], TTV-DNAemia monitoring may provide additional insight into immunological characteristics of this subgroup and could help identify patients who may warrant closer monitoring in future prospective studies.

With TTV-DNA plasma load monitoring becoming an increasingly recognized immunological biomarker tool, several multicentre interventional trials (TTVguideIT [48], TAOIST [49]) are currently evaluating the outcomes of TTV-guided dosing of immunosuppression in RTRs. In light of our findings, future research should prospectively explore the association between PTDM and TTV-DNAemia in kidney transplant recipients. Such studies should incorporate standardized PTDM screening protocols, ideally including an OGTT as the diagnostic gold standard. Furthermore, it would be valuable to investigate the longitudinal dynamics of TTV-DNAemia in patients with PTDM, and to determine whether TTV monitoring may help identify individuals at increased risk of infectious complications within this subgroup. Finally, future studies should assess whether improvements in glycaemic control are accompanied by corresponding changes in TTV-DNAemia, thereby providing further insight into the interplay between glucose metabolism and TTV replication, and whether alterations in TTV plasma load occur immediately following changes in glycaemic status or only after prolonged exposure to hyperglycaemia.

Our retrospective study has limitations. First, a certain variability in sampling time points exists. However, TTV-DNA plasma loads peaked as expected between 3 and 6 months after transplantation, and all patients developed substantial TTV-DNAemia, supporting the validity and plausibility of our viral load cohort data [9, 50]. Moreover, due to the retrospective design, diagnosis of PTDM had to rely on HbA1c values and fasting plasma glucose, which—although consistently available in our cohort—may underestimate the number of patients with PTDM [28]. In particular, HbA1c may be affected by factors such as anaemia, renal function and erythrocyte turnover especially during the early post-transplant period. Finally, although we adjusted for major confounders such as immunosuppression and age, residual confounding factors cannot be excluded in our relatively small cohort, given the multifactorial nature of PTDM, as discussed above.

### Conclusions

We describe for the first time an association between PTDM and increased TTV-DNA plasma loads during the first year after kidney transplantation. This association persisted after adjustment for major treatment-related factors and was accompanied by a trend towards more opportunistic infections in patients with PTDM. Future prospective studies are needed to further elucidate the mechanisms underlying the association between PTDM and TTV and to determine its clinical relevance.

**Table 1:** : Patient characteristics and outcomes of the overall RTR cohort of this retrospective study

Variable	Overall (n = 93)	PTDM (n = 17)	Without DM (n = 65)	*p*-value
**Demographic and clinical data**				
Male sex	61.3% (57/93)	70.6% (12/17)	55.4% (36/65)	0.2
Age (years)	55 [40 – 65]	62 [57-66]	51 [36-62]	**0.01**
BMI (kg/m²)	24 [21.2 – 27.3]	24.0 [21.6-26.8]	23.8 [20.9-27.5]	0.82
Diabetes status				
-No diabetes mellitus	69.9% (65/93)	NA	NA	NA
-PTDM	18.3% (17/93)	NA	NA	NA
-Type 1 diabetes mellitus	2.2% (2/93)	NA	NA	NA
-Type 2 diabetes mellitus	9.7% (9/93)	NA	NA	NA
Fasting plasma glucose (mg/dl)				
-at baseline	88 [80 – 98]	101 [91–114]	90 [82–94]	**0.01**
-after 3 months	96 [85 – 109]	106 [94–149]	95 [84–104]	**<0.001**
-after 6 months	96 [86 – 111]	119 [94–145]	96 [90–100]	**<0.001**
-after 12 months	95 [86 – 108]	108 [89–117]	95 [86–101]	**0.041**
HbA1c (%)				
-at baseline	5.4 [5.0 – 5.8]	5.4 [5.2 – 5.6]	5.5 [5.0 – 5.6]	0.73
-after 3 months	5.6 [5.1 – 6.3]	6.5 [6.2 – 6.7]	5.5 [5.1 – 5.7]	**<0.001**
-after 6 months	5.8 [5.3 – 6.5]	6.5 [5.8 – 7.3]	5.8 [5.2 – 6.0]	**<0.001**
-after 12 months	5.8 [5.4 – 6.3]	6.0 [5.7 – 6.0]	5.6 [5.4 – 6.0]	**0.003**
**Transplantation data**				
Number of transplantations				
-First	83.9% (78/93)	94.1% (16/17)	83.1% (54/65)	0.23
-Second	14.0% (13/93)	5.9% (1/17)	13.8% (9/65)	0.34
-Third or more	2.2% (2/93)	0% (0/17)	3.1% (2/65)	-
Deceased donor transplantation	73.1% (68/93)	82.4% (14/17)	67.7% (44/65)	0.19
Combined transplantation	6.5% (6/93)	5.9% (1/17)	7.7% (5/65)	0.64
Induction therapy				
-Basiliximab	52.7% (49/93)	64.7% (11/17)	47.7% (31/65)	0.28
-ATG	47.3% (44/93)	35.3% (6/17)	52.3% (34/65)	0.28
Warm ischemia time (h)	0.5 [0.5 – 0.7]	0.5 [0.4–0.6]	0.5 [0.5–0.7]	0.62
Cold ischemia time (h)	8.0 [3.4 – 12.0]	7.7 [6.2–14.7]	8.0 [2.8–11.7]	0.25
**Outcome data**				
Delayed graft function	21.5% (20/93)	29.4% (5/17)	21.5% (14/65)	0.35
Death (any cause)	2.2% (2/93)	5.9% (1/17)	1.5% (1/65)	-
Graft loss (death-censored)	3.2% (3/93)	5.9% (1/17)	3.1% (2/65)	-
eGFR in ml/min/1.73 m^2^				
-at baseline	15 [8 – 33]	9 [6–16]	17 [11–41]	0.08
-after 3 months	43 [30 –54]	39 [25–50]	45 [33–55]	0.46
-after 6 months	44 [30 – 58]	41 [20–60]	49 [34–57]	0.73
-after 12 months	50 [30.0 – 59]	44 [18–60]	51 [34–59]	0.66
Tacrolimus trough level (ng/ml)				
-after 3 months	9.8 [8.6 – 12.0]	9.8 [8.6-11.9]	9.8 [8.4-12.1]	0.9
-after 6 months	9.2 [7.1 – 10.5]	9.8 [7.5-11.8]	8.2 [7.0-10.5]	0.19
-after 12 months	8.2 [6.4 – 9.8]	8.9 [8.0-11.1]	7.7 [6.2-9.2]	0.074
Cumulative prednisolone dose (mg)				
-after 3 months	1705 [1663 – 1883]	1665 [1615-1780]	1720 [1675-2405]	0.21
-after 6 months	2213 [2129 – 2717]	2195 [2133-2580]	2228 [2130-2855]	0.73
-after 12 months	3108 [3030 – 3618]	3120 [3058-3623]	3153 [3040-3940]	0.85
Number of steroid pulses				
-no pulse	77.4% (72/93)	76.5% (13/17)	75.4% (49/65)	0.6
-1 pulse	19.4% (18/93)	17.6% (3/17)	21.5% (14/65)	0.51
-≥2 pulses	3.2% (3/93)	5.9% (1/17)	3.1% (2/65)	-
Rejection	10.8% (10/93)	17.6% (3/17)	10.8% (7/65)	0.34
-T-cell mediated	8.6% (8/93)	17.6% (3/17)	9.2% (6/65)	0.6
-Antibody-mediated	1,1% (1/93)	0% (0/0)	1.5% (1/65)	-
Infectious complications	25.8% (24/93)	35.3% (6/17)	23.1% (15/65)	0.23
-BKV replication	12.9% (12/93)	17.6% (3/17)	10.8% (7/65)	0.31
-CMV replication	7.5% (7/93)	11.8% (2/17)	7.7% (5/65)	0.4
-EBV replication	1.1% (1/93)	0.0% (0/17)	1.5% (1/65)	-
-*Aspergillus* spp. infection	1.1% (1/93)	0.0% (0/17)	1.5% (1/65)	-
-*Candida* spp. infection	2.2% (2/93)	5.9% (1/17)	0.0% (0/65)	-
-Hepatitis E virus infection (prolonged)	1.1% (1/93)	0.0% (0/17)	1.5% (1/65)	-

Data are presented as percentages (absolute numbers) or median [interquartile range]. Group sizes are indicated in parentheses. PTDM status refers to patients who developed PTDM at any time during the first year after transplantation. Comparisons between patients with PTDM and patients without DM were performed using the Chi-square test for categorical variables and the Mann–Whitney U test for continuous variables. P-values <0.05 were considered statistically significant, and statistically significant findings are shown in bold. NA = not applicable. Statistical testing was not performed for outcomes with very low event numbers; corresponding table entries are shown as a dash (–)

**Table 2:** Linear mixed model results for predictors of TTV-DNA plasma loads after transplantation

**Fixed effects**	**Estimate**	**95% CI**	***p*** **-value**
*Intercept*	1.51	-0.15–3.16	0.075
*Time*	0.21	0.15–0.26	< 0.001
*Tacrolimus level*	0.10	0.04–0.16	0.001
*HbA1c*	0.365	0.081–0.65	0.012
**Random effects**	**Estimate**	**95% CI**	***p*** **-value**
*Intercept*	0.00		
*Residual*	3.84	3.24–4.55	< 0.001

Estimated fixed and random effects from the linear mixed-effects model assessing predictors of longitudinal TTV-DNAemia after kidney transplantation. Fixed effects included time after transplantation, tacrolimus trough level, and HbA1c. Estimates are presented with 95% confidence intervals (95% CI)

**Fig. 3:**
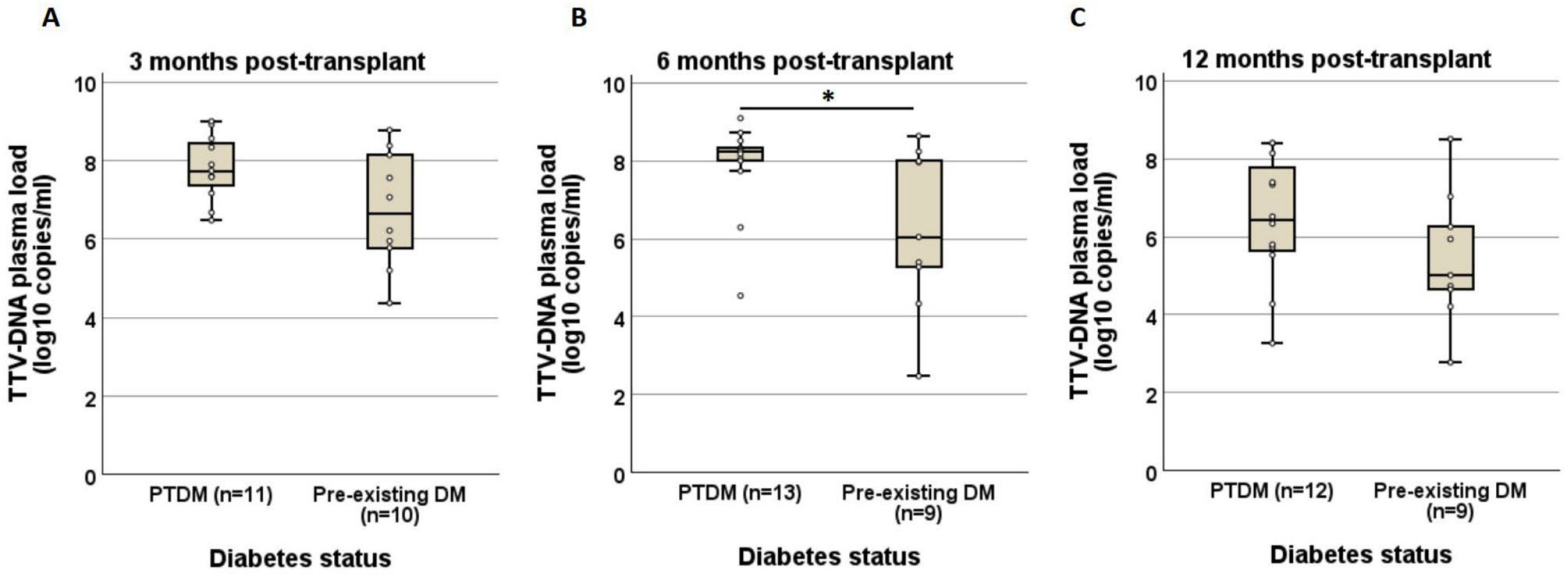
**A**-**C**. Comparison of Torque teno virus (TTV)-DNA plasma load of patients with post-transplant diabetes mellitus (PTDM) and patients with pre-existing diabetes mellitus (DM) at 3 months (**A**), 6 months (**B**), and 12 months (**C**) after kidney transplantation. TTV-DNA plasma loads are presented as log₁₀ copies/ml. Patients were classified as PTDM if the diagnosis had been established at or before the respective time point. Boxplots represent median values and interquartile ranges, while individual data points depict the distribution of available measurements. The number of available samples for each group is indicated below the respective boxplots. Differences in TTV-DNAemia levels between groups were assessed using the Mann–Whitney U test

## Supplementary Information

Below is the link to the electronic supplementary material.

Supplementary Material 1

## Data Availability

The datasets generated or analyzed during this study are available from the corresponding author on reasonable request.

## Abbreviations

AIC: Akaike’s Information Criterion
ATG: Anti-thymocyte globulin
BKV: BK virus
BMI: Body mass index
CMV: Cytomegalovirus
DM: Diabetes mellitus
EBV: Epstein–Barr virus
eGFR: Estimated glomerular filtration rate
IQR: Interquartile range
OGTT: Oral glucose tolerance test
PTDM: Post-transplantation diabetes mellitus
TTV: Torque teno virus
