# Supplementary material for: Association between Torque teno virus-DNA plasma loads and post-transplantation diabetes mellitus in the first year after kidney transplantation
Source: BMC Nephrol. 2026 Jul 9;27:410. doi: 10.1186/s12882-026-05192-6 (PMC13355353; doi:10.1186/s12882-026-05192-6)
Supplement: Supplementary file 1 — Supplementary Material 1 [file 12882_2026_5192_MOESM1_ESM.docx]

**Supplementary Data**

- Supplementary Figure S1: Study flow chart
- Supplementary Figure S2: Dynamics of TTV-DNA plasma load over time including longitudinal trajectories for the whole cohort
- Supplementary Figure S3: TTV-DNA plasma load in patients with and without pre-existing diabetes mellitus at different time points after transplantation.
- Supplementary Table 1: Glycemic parameters in post-transplantation diabetes mellitus versus pre-existing diabetes mellitus
- Supplementary Table 2: Multiple linear regression model evaluating tacrolimus though level as a confounding variable
- Supplementary Table 3: Estimated effects of age on TTV-DNAemia based on linear regression analysis

**Supplementary Figure S1**


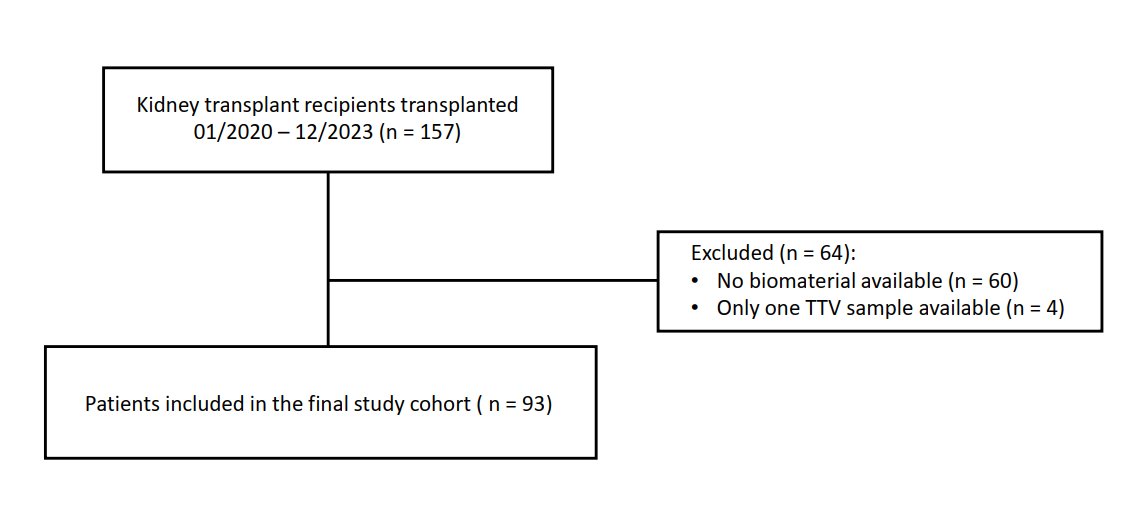


**Supp. Fig S1. Study flow chart.** Overview of patient selection and reasons for exclusion. A total of 157 kidney transplant recipients transplanted between January 2020 and December 2023 at our transplant center were screened for inclusion. 64 patients were excluded due to unavailable biomaterial for TTV-PCR analysis (n = 60) or because only one TTV result was available for subsequent analyses(n = 4). The final study cohort included 93 patients.

**Supplementary Figure S2**

**
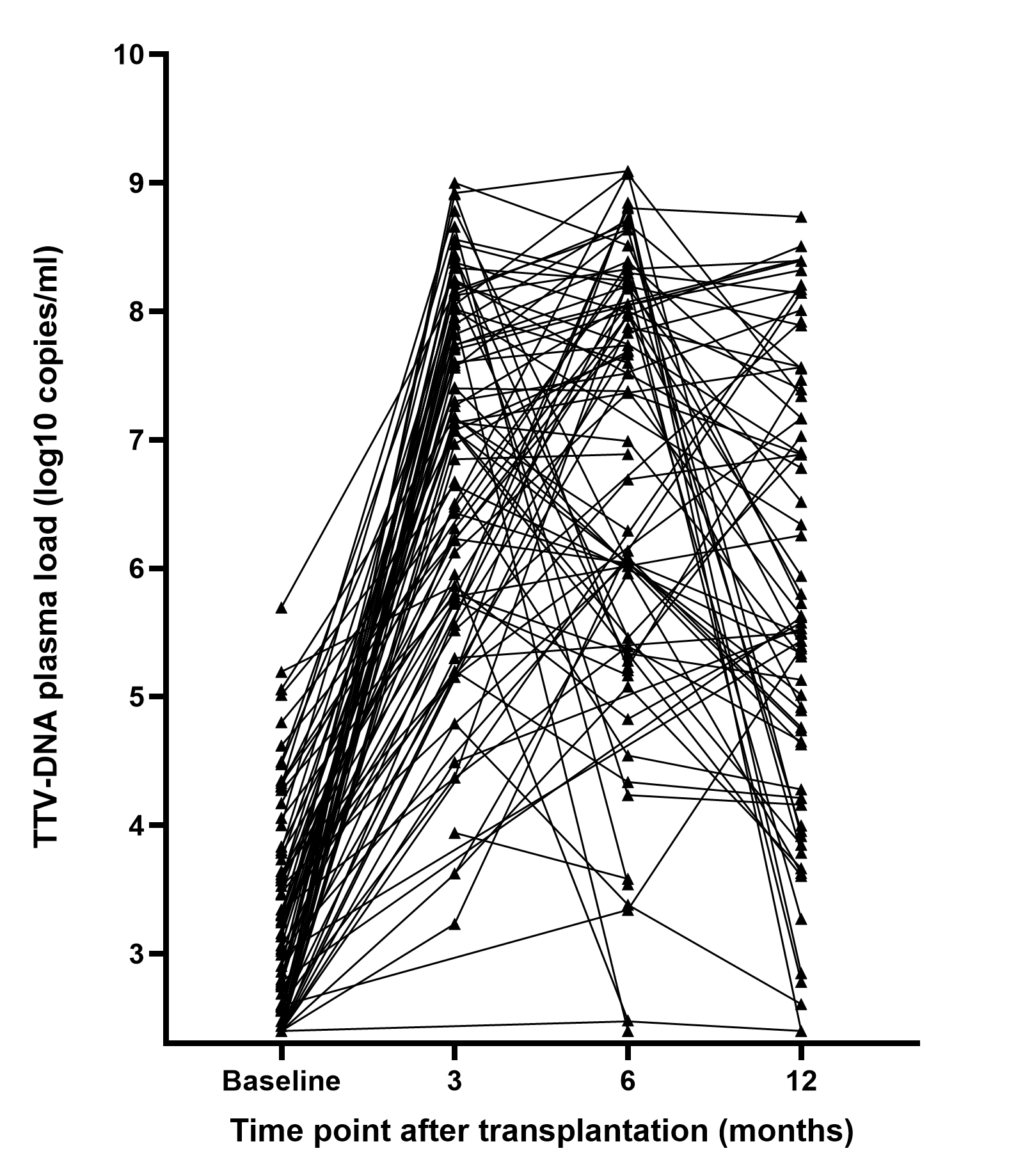
**

**Suppl. Fig. S2. Dynamics of TTV-DNA plasma load over time including longitudinal trajectories.** Illustration of TTV-DNA plasma loads at baseline and 3, 6, and 12 months after kidney transplantation in the complete study population (n = 93). Individual symbols represent TTV-DNA plasma loads expressed as log₁₀ copies/mL. Individual longitudinal trajectories are displayed by connecting measurements obtained from the same patient across different time points.


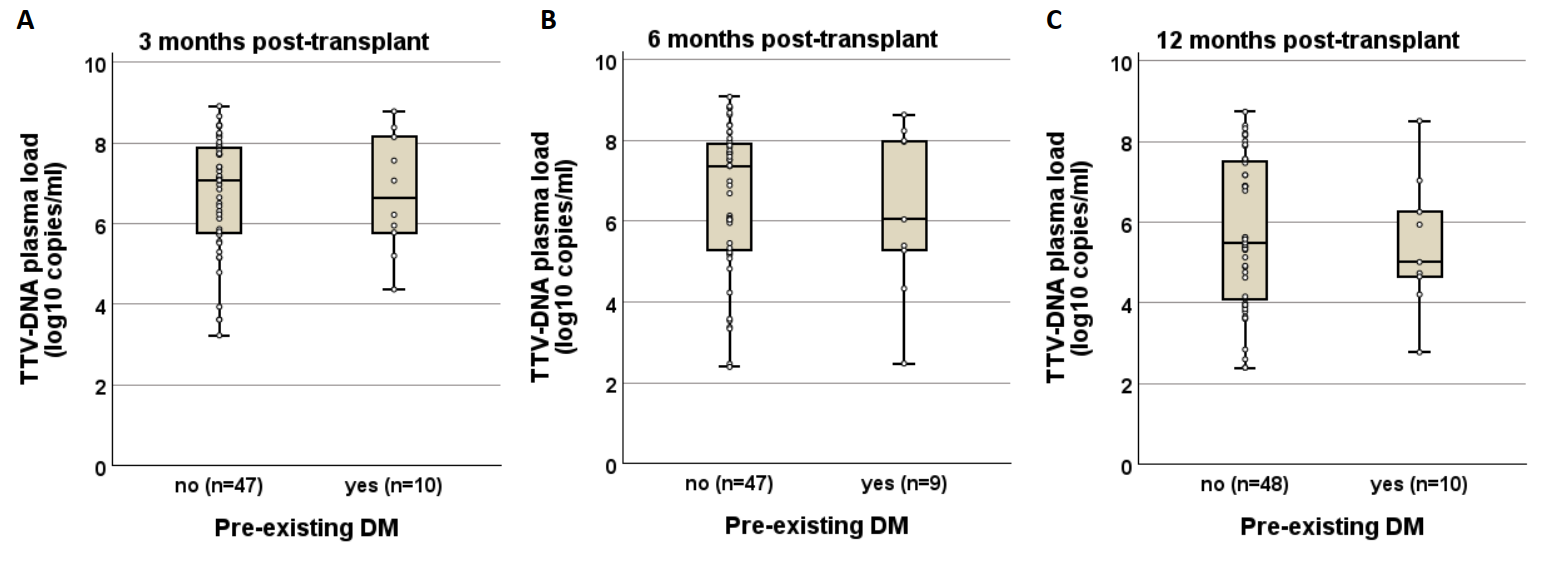
**Supplementary Figure S3**

**Supp. Fig S3 A-C. TTV-DNA plasma load in patients with and without pre-existing diabetes mellitus at different time points after transplantation.** Torque teno virus (TTV)-DNA plasma load in patients with and without pre-existing diabetes mellitus (DM) at 3 months (A), 6 months (B), and 12 months (C) after kidney transplantation. Patients with pre-existing DM included individuals with pre-existing type 1 or type 2 diabetes mellitus. Patients with PTDM were excluded from this analysis. TTV-DNA plasma loads are presented as log₁₀ copies/ml. Boxplots represent median values and interquartile ranges, while individual data points depict the distribution of available measurements. The number of available samples for each group is indicated below the respective boxplots. Differences in TTV-DNAemia between groups were assessed using the Mann–Whitney U test. No significant differences in TTV-DNA plasma loads were observed between patients with and without pre-existing DM at any time point.

**Supplementary Table 1: Glycemic parameters in post-transplantation diabetes mellitus versus pre-existing diabetes mellitus**

|  | **PTDM** | **Pre-existing DM** | **p-value** |
| --- | --- | --- | --- |
| Fasting plasma glucose (mg/dl) |  |  |  |
| - after 3 months | 105 [98-129] | 119 [113-138] | 0.39 |
| - after 6 months | 120 [96-140] | 110 [85-146] | 0.79 |
| - after 12 months | 108 [97-117] | 143 [126-148] | **0.016** |
| HbA1c (%) |  |  |  |
| - after 3 months | 6.6 [6.2-6.8] | 7.0 [6.6-7.3] | 0.07 |
| - after 6 months | 6.7 [6.3-7.6] | 6.7 [6.6-6.8] | 0.74 |
| - after 12 months | 6.3 [5.9-6.5] | 7.3 [6.6-7.7] | **0.003** |

Fasting plasma glucose and HbA1c levels are shown for patients with post-transplant diabetes mellitus (PTDM) and patients with pre-existing diabetes mellitus (DM) at 3, 6, and 12 months after kidney transplantation. Patients were classified as PTDM if the diagnosis had been established at or before the respective time point. Data are presented as median values with interquartile ranges (IQR). Group comparisons were performed at each time point using the Mann–Whitney U test. Statistically significant p-values (p <0.05) are highlighted in bold. No significant differences in glycaemic parameters were observed between groups at 3 and 6 months after transplantation. However, at 12 months, patients with pre-existing DM showed significantly higher fasting plasma glucose and HbA1c levels compared with patients with PTDM.

**Supplementary Table 2: Multiple linear regression model evaluating tacrolimus trough level as a confounding variable**

| **At 3 months** | **Estimate** | **Standard error** | **t-value** | **p-value** |
| --- | --- | --- | --- | --- |
| *Intercept* | 6.23 | 0.49 | 12.78 | **<0.001** |
| *PTDM status* | 1.13 | 0.46 | 2.45 | **0.02** |
| *Tacrolimus level* | 0.04 | 0.04 | 1.03 | 0.32 |
| **At 6 months** | **Estimate** | **Standard error** | **t-value** | **p-value** |
| *Intercept* | 6.16 | 0.66 | 9.41 | **<0.001** |
| *PTDM status* | 1.38 | 0.58 | 2.38 | **0.02** |
| *Tacrolimus level* | 0.06 | 0.07 | 0.88 | 0.38 |

A multiple linear regression model was used to evaluate whether tacrolimus trough levels confounded the association between post-transplant diabetes mellitus (PTDM) and TTV-DNA plasma loads at 3 and 6 months after kidney transplantation. The dependent variables were TTV-DNA plasma load at either 3 months or 6 months, while the independent variables included PTDM status and the tacrolimus trough levels at the respective time points. PTDM status remained significantly associated with higher TTV-DNA plasma loads after inclusion of tacrolimus trough levels as a potential confounding variable. Statistically significant p-values (p-value <0.05) are highlighted in bold.

**Supplementary Table 3: Estimated effects of age on TTV-DNA plasma load based on linear regression analysis**

|  | **Age** | **Predicted TTV-DNA load (log₁₀ copies/ml)** |
| --- | --- | --- |
| **PTDM group** | 62 [57-66] | 5.68 [5.58-5.76] |
| **Without DM** | 51 [36-62] | 5.46 [5.16-5.58] |
| **Difference** | 11 | 0.22 |

To assess the potential influence of age on TTV-DNAemia levels, a linear regression model was calculated with TTV-DNA plasma loads as the dependent variable and age as the independent variable. The resulting regression equation was:

$$TTV level (log₁₀ copies/ml) =4.44 +0.02 \times Age (years)$$

This equation was then used to estimate age-related effects on TTV-DNAemia for each time point. For this purpose, the median age of the post-transplant diabetes mellitus (PTDM) group and the median age of the non-PTDM group at 3, 6, and 12 months were added to the equation to calculate the corresponding predicted TTV-DNA plasma loads. In the table, the last row represents the difference between the median values of patients with PTDM and without diabetes mellitus (DM).
